# Structural Modification and Enhanced Gel Properties of Peanut Protein via Co-Precipitation with Egg White Protein

**DOI:** 10.3390/foods15122187

**Published:** 2026-06-17

**Authors:** Xiaoyu Liu, Ming Zhang, Manqi Yang, Cui Han, Yuxi Shen, Yujie Su, Yining Zhang, Yuanqi Lv

**Affiliations:** 1 College of Food Science and Technology, Henan University of Technology, Zhengzhou 450001, China; 2 Food Research Center, Zhongyuan Institute, Zhejiang University, Zhengzhou 450001, China; 3 School of Food Science and Technology, Jiangnan University, Wuxi 214122, China; 4 School of Food Engineering, Henan Polytechnic, Zhengzhou 450000, China

**Keywords:** peanut protein, egg white protein, co-precipitated protein, structural properties, gel properties

## Abstract

Peanut protein (PP) is an abundant plant protein resource with limited gelation performance. In this study, the effects of co-precipitation with egg white protein (EWP) on the structural and gelation properties of PP were investigated. Structural analysis revealed that co-precipitation induced secondary structure rearrangement of PP, accompanied by decreased *α*-helix and *β*-sheet contents and increased random coil and *β*-turn contents. These changes were associated with the exposure of hydrophilic groups and the partial shielding of hydrophobic regions, contributing to the significantly improved solubility of PP-EWP co-precipitated proteins (*p* < 0.05). These structural changes were conducive to the formation of a denser and more continuous gel network. Compared with the PP gel, the gel prepared from PP-EWP co-precipitated protein at the PP:EWP ratio of 2:1 showed an increase in gel strength from 429.30 g to 911.94 g and in water holding capacity from 56.78% to 85.53%. This study provides a theoretical basis and practical guidance for improving the gel properties of PP through co-precipitation and developing functional peanut protein ingredients, although the relatively high cost of EWP should be considered in practical applications.

## 1. Introduction

In recent years, plant proteins have emerged as important components in the development of healthy and sustainable foods due to their high nutritional value and relatively low environmental and resource impacts [1]. Peanut protein (PP) is a high-quality plant protein source with nutritional value comparable to that of animal proteins [2]. However, the application of PP in the food industry has been limited by its poor gelation properties [3,4]. From a structural perspective, the compact conformation and aggregation tendency of PP restrict the exposure of reactive groups and reduce the efficiency of intermolecular interactions required for the formation of stable gel networks [5]. Therefore, developing an effective modification strategy to regulate the structure of PP is important for improving its gelation performance and broadening its application in food systems.

Co-precipitation has emerged as an innovative approach that enables simultaneous protein reassembly and modification throughout extraction [6]. This technology generally involves pH adjustment to induce protein unfolding, intermolecular interactions, and reassembly, thereby regulating protein structure and related functional properties [1,7]. Protein co-precipitates have been reported to improve the texture, stability, nutritional characteristics, and ingredient applicability of food systems [8]. These effects have been demonstrated in different co-precipitated protein systems. Previous research has demonstrated that co-assembly of soy protein isolate (SPI) and wheat protein (WP) promoted structural interactions between the two proteins and significantly increased the solubility of WP from 5% to 72% without disrupting its primary structure [9]. Similarly, soybean-wheat co-precipitated proteins exhibited significantly improved water dispersibility, as well as enhanced gel strength and water holding capacity, indicating that co-precipitation may improve both dispersion behavior and gelation performance [1]. In addition, pea–grass carp co-precipitated dual-protein systems prepared under suitable alkaline extraction conditions showed improved solubility and gel properties [10]. These studies indicate that co-precipitation has been explored in different dual-protein systems, including plant–plant and plant–animal protein combinations, and can regulate protein structure and improve functional performance in co-precipitated protein systems.

Egg white protein (EWP) is composed of ovalbumin, ovotransferrin, ovomucin, lysozyme, and other constituents [11]. Its molecular structure, in which hydrophobic residues are buried in the interior while hydrophilic residues are mainly exposed on the surface, confers high solubility [12]. Furthermore, EWP contains abundant sulfhydryl groups, which can form disulfide bonds during gelation, promoting the development of gel networks [13]. These structural and functional features suggest that EWP may serve as a suitable exogenous protein component for modifying PP through co-precipitation and may contribute to the conformational rearrangement and heat-induced gel network formation of PP. However, how co-precipitation with EWP alters the conformation of PP and regulates subsequent gel network formation remains unclear. Therefore, this study investigated the structural changes and gelation behavior of co-precipitated proteins prepared from PP and EWP, with particular attention to conformational changes, intermolecular interactions, and heat-induced gel network formation. This study may provide a theoretical basis for modifying PP through co-precipitation and expanding its potential use as a functional plant protein ingredient with enhanced gelation properties.

## 2. Materials and Methods

### 2.1. Materials

Low-temperature cold-pressed peanut meal (protein content 45.8%) was purchased from Anyang Tianxiangrui Food Technology Co., Ltd. (Anyang, China). EWP (protein content 80.2%) was provided by Anhui Rongda Food Co., Ltd. (Guangde, China). All chemicals used were of analytical grade and purchased from Shanghai Macklin Biochemical Co., Ltd. (Shanghai, China) and Tianjin Comio Chemical Reagent Co., Ltd. (Tianjin, China). Distilled water was used throughout the study.

### 2.2. Preparation of Co-Precipitated Proteins

Peanut meal and egg white protein powder were mixed at PP:EWP ratios of 6:1, 4:1, and 2:1 (*w*/*w*) and dispersed in distilled water at a ratio of 1:10 (*w*/*v*). The pH was adjusted to 10.0 using 2 mol/L NaOH, and the mixture was stirred in a water bath at 50 °C for 30 min. After centrifugation at 5000× *g* for 10 min using a high-speed refrigerated centrifuge (TGL-16M, Xiangyi Centrifuge Instrument Co., Ltd., Changsha, China), the supernatant was collected. The pH of the supernatant was then adjusted to 4.5 using 0.5 mol/L HCl, and the mixture was allowed to stand at 4 °C for 24 h. The precipitate was collected after centrifugation at 5000× *g* for 10 min, redispersed in an appropriate volume of distilled water, and neutralized to pH 7.0 with 2 mol/L NaOH. Finally, the co-precipitates were obtained by freeze-drying for 48 h using a freeze dryer (LGJ-25C, Sihuan Furuikeyi Technology Development Co., Ltd., Beijing, China). They were named as Co6:1, Co4:1 and Co2:1, respectively. Peanut protein (PP) extracted from peanut meal using the same procedure served as the control.

### 2.3. Determination of Protein Solubility

A protein solution (1.5 mg/mL) was prepared in phosphate-buffered saline (PBS, 0.01 mol/L, pH 7.4). The total protein content was determined by the biuret method at 540 nm using a UV–Vis spectrophotometer (UV-6100S, Mapada Instruments Co., Ltd., Shanghai, China). After centrifugation at 5000× *g* for 10 min, the supernatant was collected, and the soluble protein content was determined by the Bradford method at 595 nm using a microplate reader (Varioskan Lux, Thermo Fisher Scientific, Shanghai, China). All measurements were performed in triplicate. The solubility was calculated using the following formula:

(1) $$Solubility\;(\%)=\frac{C}{C_{0}}\times 100$$

*C* is the soluble protein concentration (mg/mL) in the supernatant, and *C*_0_ is the total protein concentration (mg/mL).

### 2.4. Determination of Free Sulfhydryl Content (SH_F_)

Ellman’s reagent was prepared by dissolving DTNB in Tris-glycine buffer to a concentration of 4 mg/mL. Then, 2.5 mL of Tris-glycine buffer (0.086 mol/L Tris, 0.09 mol/L glycine, 0.004 mol/L EDTA, pH 8.0) was mixed with 0.5 mL of diluted protein solution, followed by the addition of 20 μL of Ellman’s reagent. The resulting mixture was reacted in the dark for 15 min and then the absorbance was measured at 412 nm using a UV–Vis spectrophotometer (UV-6100S, Mapada Instruments Co., Ltd., Shanghai, China) [14]. The free sulfhydryl content was calculated as follows:

(2) $$SH_{F}\;(\mu \mathrm{mol}/g)=\frac{73.53\times A_{412}\times D}{C}$$

*C* is the protein concentration (mg/mL), *D* is the dilution factor, *A*_412_ is the absorbance of the sample, and 13,600 is the molar extinction coefficient.

### 2.5. Determination of Particle Size and Zeta Potential

A nanoparticle size and zeta potential analyzer (BeNano 90 Zeta, Bettersize Instruments Co., Ltd., Dandong, China) was used to determine the particle size and zeta potential at 25 °C. The protein samples were diluted to a final concentration of 0.5 mg/mL with 0.01 mol/L PBS (pH 7.4). The refractive indices of the protein and the dispersant were set to 1.450 and 1.330, respectively [15].

### 2.6. Determination of Surface Hydrophobicity (H_0_)

The protein solutions were diluted to concentrations of 0.04, 0.06, 0.08, 0.10, and 0.15 mg/mL. Then, 4 mL of each diluted protein solution was mixed with 25 μL of ANS (8 mmol/L) solution and reacted for 10 min in the dark. The fluorescence intensity of the mixtures was measured using a fluorescence spectrophotometer (FL970 Plus, Hitachi High-Tech Corporation, Tokyo, Japan) at excitation and emission wavelengths of 390 nm and 470 nm, respectively [16]. The slope of the linear regression equation between fluorescence intensity and protein concentration was defined as H_0_.

### 2.7. Fluorescence Spectrum

The emission spectra of the samples (0.2 mg/mL) were recorded from 300 to 400 nm using a fluorescence spectrophotometer (FL970 Plus, Hitachi High-Tech Corporation, Tokyo, Japan). The measurement was performed with an excitation wavelength of 280 nm and a scan speed of 60 nm/min. Both the excitation and emission slit widths were set at 5.0 nm [17].

### 2.8. Fourier Transform Infrared Spectroscopy (FTIR) Measurement

The freeze-dried sample powders were mixed with KBr at a mass ratio of 1:100 and then compressed into thin films. The films were scanned using a Fourier transform infrared spectrometer (Nicolet 6700FT-IR, Thermo Fisher Scientific Inc., Waltham, MA, USA). A total of 32 scans were performed over the range of 4000 to 500 cm^−1^ at a resolution of 4 cm^−1^. The secondary structure of the amide I band (1700–1600 cm^−1^) was analyzed using Peak Fit Version 4.12 software [18].

### 2.9. The Microstructure of Proteins

The protein powders were observed using a scanning electron microscope (SU 8200, Hitachi High-Tech Corporation, Tokyo, Japan) at a magnification of ×2000.

### 2.10. Sodium Dodecyl Sulfate-Polyacrylamide Gel Electrophoresis (SDS-PAGE)

The protein samples were diluted to 1 mg/mL with 0.01 mol/L PBS (pH 7.4) and mixed with 5× SDS sample buffer at a ratio of 4:1 (*v*/*v*). The mixtures were then heated in a boiling water bath for 3 min. Then, 12.5 μL of each sample was loaded into the gel lanes, and a protein marker (11–180 kDa) was used to estimate the molecular weights of the protein bands. Electrophoresis was carried out at 80 V in the concentrate gel and 120 V in the separation gel. After electrophoresis, the gel was stained with Coomassie Brilliant Blue R-250 solution (0.05%, *w*/*v*) for 30 min and destained with a solution of methanol, acetic acid, and deionized water (25:7:68, *v*/*v*/*v*) [15].

### 2.11. Molecular Docking

Molecular docking analysis was performed using Discovery Studio 2019 Client (BIOVIA, San Diego, CA, USA). The crystal structures of ovalbumin (PDB: 1OVA), the major region of arachin from PP (PDB: 3C3V), and the major region of conarachin from PP (PDB: 3SMH) were obtained from the Protein Data Bank. The docking simulation was performed according to a previously reported method [19]. Because the interaction sites between the proteins were not known in advance, docking was performed to explore the possible interaction modes between the proteins. The docking results were used to analyze the possible intermolecular interactions at the binding interface, including hydrogen bonding, electrostatic interactions, and van der Waals interactions. The docking conformations were visualized using PyMOL 2.5 and Discovery Studio 2019.

### 2.12. Preparation of the Protein Gels

The samples were dissolved in 0.01 mol/L PBS (pH 7.4) to prepare protein solutions at a concentration of 15% (*w*/*v*). The solutions were then heated at 90 °C for 30 min and rapidly cooled to form gel samples (GPP, GCo6:1, GCo4:1, and GCo2:1). The resulting gels were stored at 4 °C for 24 h before further analysis [1].

### 2.13. Determination of Gel Strength

The gel properties of the samples were evaluated using a texture analyzer (TA.XTplusC, Stable Micro Systems Ltd., Godalming, UK) equipped with a P/0.5 probe. During the test, the pre-test, test, and post-test speeds were set at 2 mm/s, 1 mm/s, and 2 mm/s, respectively, with a test distance of 10 mm and a trigger force of 1 g [20].

### 2.14. Determination of Water Holding Capacity (WHC)

The WHC of the gels was determined by a centrifugation method [21]. Gel samples (5 g) were wrapped with two layers of filter paper and placed in centrifuge tubes, and the total mass was recorded. Subsequently, the samples were centrifuged at 4 °C and 5000× *g* for 10 min. The tubes containing the gel samples were weighed again. WHC was calculated as follows:

(3) $$WHC\;(\%)=\frac{m_{2}-m_{0}}{m_{1}-m_{0}}$$

In the formula: *m*_0_ is the mass (g) of the centrifuge tube; *m*_1_ is the total mass (g) of the gel and the tube before centrifugation; *m*_2_ is the total mass (g) of the gel and the tube after centrifugation.

### 2.15. Intermolecular Forces of the Protein Gels

The following extraction solutions were prepared: A1 (0.6 mol/L NaCl), A2 (0.6 mol/L NaCl + 1.5 mol/L urea), A3 (0.6 mol/L NaCl + 8 mol/L urea), and A4 (0.6 mol/L NaCl + 8 mol/L urea + 0.5 mol/L *β*-ME, pH 7.0). Gel samples (0.5 g) were mixed with 5 mL of A1 extraction solution and homogenized at 5000 rpm for 3 min. The mixtures were then centrifuged at 5000× *g* and 4 °C for 15 min. The supernatants (designated as S1) were collected and stored at 4 °C. An aliquot (5 mL) of A2 extraction solution was added to the centrifuge tubes containing the precipitates, and the above steps were repeated for the A3 and A4 extraction solutions. Each supernatant was mixed with 4.5 mL of 20% trichloroacetic acid and allowed to precipitate at 4 °C for 1 h, followed by centrifugation at 5000× *g* for 10 min to obtain the protein precipitate. The resulting protein precipitate was re-dissolved in 2 mL of 1 mol/L NaOH. Protein concentration in the supernatants was determined using the Coomassie Brilliant Blue method. The relative protein contents in S1, S2, S3, and S4 were used to estimate the contributions of ionic bonds, hydrogen bonds, hydrophobic interactions, and disulfide bonds, respectively, to the gel systems [22].

### 2.16. The Microstructure of the Protein Gels

The gel samples were sliced into thin sections (5 × 5 × 1.5 mm^3^) and fixed in 2.5% glutaraldehyde (0.1 mol/L PBS, pH 7.2) for 24 h. The gel slices were then rinsed with PBS (0.1 mol/L, pH 7.2) and dehydrated through a graded series of ethanol concentrations. After freezing at −40 °C for 2 h, the gel slices were freeze-dried. The microstructure of the gels was examined using a scanning electron microscope (SU 8200, Hitachi High-Tech Corporation, Tokyo, Japan) at a magnification of ×20,000 [23].

### 2.17. Statistical Analysis

All experiments were performed in triplicate, and the results were expressed as mean ± standard deviation (SD). Data were analyzed by one-way analysis of variance (ANOVA) using IBM SPSS Statistics 27 software, followed by Duncan’s multiple range test to determine significant differences among samples at *p* < 0.05. The relevant figures were generated using Origin 2021 software.

## 3. Results and Discussion

### 3.1. Protein Solubility and the Content of Free Sulfhydryl

**Figure 1:**
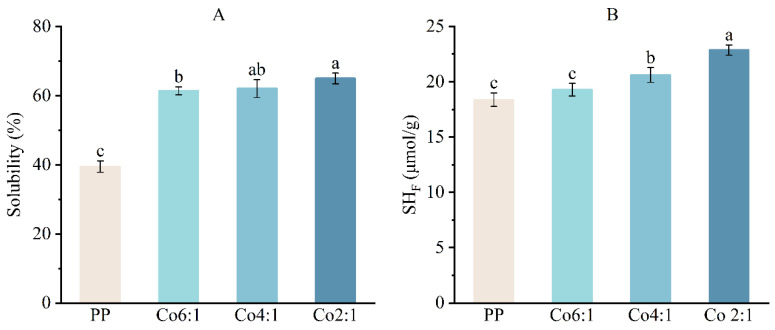
Solubility (**A**) and free sulfhydryl content (SH_F_, (**B**)) of PP and PP-EWP co-precipitated proteins with different ratios. Different letters indicate significant differences among samples (*p* < 0.05).

Protein solubility is an important indicator of protein functionality in food systems and of its practical application potential [24]. As shown in Figure 1A, the co-precipitated proteins exhibited significantly higher solubility than PP (*p* < 0.05). This may be attributed to the inherently low solubility of native PP, which is related to its compact structure and the limited exposure of hydrophilic and ionizable groups [3]. Previous studies have reported that EWP exhibits high solubility [25]. Ovalbumin, the major component of EWP, is a naturally occurring globular protein with a hydrophobic core surrounded by hydrophilic groups, which contributes to its favorable water solubility [12,26]. Co-precipitation promoted the exposure of hydrophilic groups on the protein surface through the simultaneous denaturation and precipitation of PP and EWP, thus enhancing solubility. This result was consistent with previous reports that co-precipitated SPI and WP exhibited higher solubility than the individual proteins [1].

Disulfide bonds and sulfhydryl groups are involved in dynamic exchange during co-precipitation, which is important for maintaining protein tertiary structure [7]. As displayed in Figure 1B, the PP-EWP co-precipitated proteins exhibited significantly higher SH_F_ than PP (*p* < 0.05), with a progressive increase observed as the proportion of EWP in the co-precipitates increased. This increase can be partially attributed to the relatively high sulfhydryl content of EWP [27]. Furthermore, the co-precipitation induced structural rearrangement of proteins, leading to partial unfolding and exposure of previously buried sulfhydryl groups [28]. Meanwhile, dynamic disulfide bond exchange, involving interconversion between sulfhydryl groups and disulfide bonds, may also contribute to the observed increase in SH_F_.

### 3.2. Particle Size and Zeta Potential

**Figure 2:**
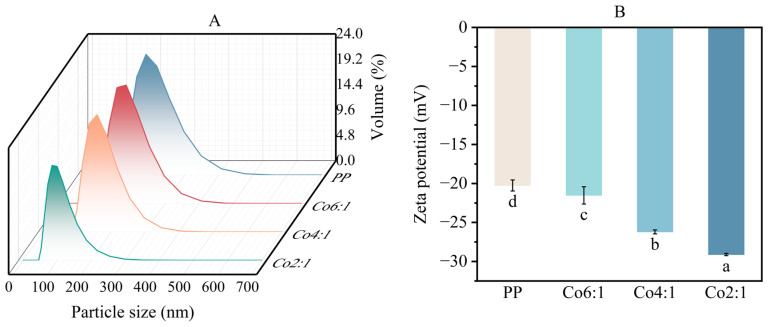
Particle size (**A**) and zeta potential (**B**) of PP and PP-EWP co-precipitated proteins at different ratios. Different letters indicate significant differences among samples (*p* < 0.05).

Particle size serves as a direct indicator of protein degradation and aggregation states [29]. As shown in Figure 2A, the particle size of PP-EWP co-precipitates gradually decreased with increasing EWP proportions. This phenomenon was primarily attributed to the conformational rearrangement of EWP and PP during co-precipitation. EWP is highly hydrophilic and contains abundant polar groups [12]. During co-denaturation and precipitation with PP, the hydrophilic groups of EWP molecules became predominantly exposed on the protein surface, thereby reducing intermolecular aggregation caused by hydrophobic interactions. Meanwhile, the significant increase in SH_F_ (Figure 1B) indicated protein unfolding and partial cleavage of disulfide bonds, which reduced steric hindrance and consequently facilitated the formation of smaller, more uniformly distributed protein aggregates. These reduced protein particle sizes promoted more homogeneous dispersion in aqueous solutions, thereby enhancing solubility [16]. This was consistent with the solubility results (Figure 1A).

The zeta potential reflects the surface charge of a molecule and the electrostatic interactions between proteins [29]. As illustrated in Figure 2B, the zeta potential values of both PP and PP-EWP co-precipitates remained negative, while the absolute values increased progressively with increasing proportions of EWP. Among the samples, Co2:1 exhibited the highest absolute zeta potential. Previous studies reported that both ovalbumin (the primary component of EWP) and PP had an isoelectric point (pI) of 4.5 [3,27]. Under the measurement conditions (pH 7.4), both proteins carried strong net negative charges. During co-precipitation, intermolecular interactions between EWP and PP promoted the preferential localization on the protein surface of EWP components rich in acidic amino acids, such as ovalbumin, thereby increasing the surface charge density of the co-precipitates [27]. Furthermore, conformational unfolding of PP induced by EWP exposed internal negatively charged groups, further enhancing the effective surface charge. The elevated absolute zeta potential values indicated enhanced stability of the co-precipitated proteins, with strengthened electrostatic repulsion effectively suppressing protein aggregation [15]. These findings were consistent with the particle size (Figure 2A) and solubility (Figure 1A) results.

### 3.3. Surface Hydrophobicity and Fluorescence Spectroscopy

**Figure 3:**
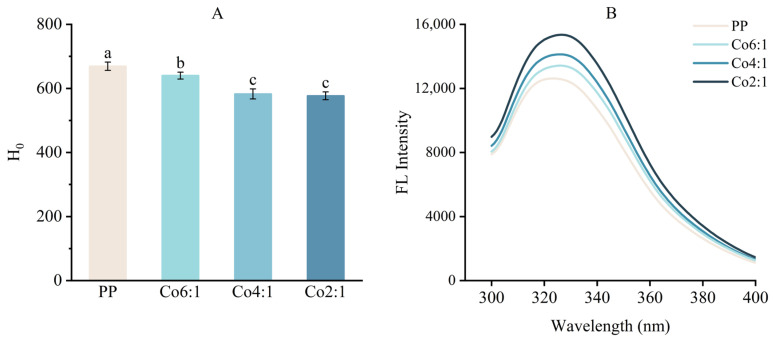
Surface hydrophobicity (**A**) and fluorescence spectra (**B**) of PP and PP-EWP co-precipitated proteins at different ratios. Different letters indicate significant differences among samples (*p* < 0.05).

Surface hydrophobicity serves as a key indicator for protein conformational changes, reflecting both the exposure of internal hydrophobic groups and their interaction with water, thereby providing insights into protein functional properties [5]. PP showed the highest surface hydrophobicity (Figure 3A) among all samples, suggesting greater exposure of hydrophobic groups on the protein surface. Increasing the proportion of EWP in the co-precipitates resulted in a decrease in measured surface hydrophobicity, suggesting that although partial unfolding has occurred during alkaline treatment, the exposed hydrophobic groups were subsequently re-buried within the co-precipitated aggregates or partially shielded by EWP. A similar decrease in surface hydrophobicity has also been reported in co-precipitated SPI-WP proteins and was attributed to reduced exposure of hydrophobic groups on the protein surface [1]. These effects contributed to the burial of hydrophobic residues within the protein complexes, thereby reducing surface hydrophobicity. Consequently, the decrease in surface hydrophobicity was accompanied by enhanced protein solubility (Figure 1A).

Intrinsic fluorescence spectroscopy is utilized to monitor conformational transitions with high sensitivity by detecting subtle changes in the microenvironments of tryptophan residues within protein molecules [30]. The aromatic amino acids (tryptophan, tyrosine, and phenylalanine) function as inherent fluorophores that emit characteristic fluorescence when excited at 280 nm, with tryptophan being the predominant contributor due to its high abundance and sensitivity to environmental polarity [31]. In this study, PP-EWP co-precipitated proteins were utilized to probe conformational changes. In Figure 3B, the fluorescence intensities of the co-precipitates were significantly higher than those of PP and increased progressively with higher EWP proportions. This increase was closely associated with the elevated SH_F_ (Figure 1B), as interactions between PP and EWP during co-precipitation induced partial cleavage of disulfide bonds in PP, which reduced the disulfide quenching groups originally located near tryptophan residues in the hydrophobic core [32]. The fluorescence spectra also showed a progressive red shift in the emission peak along with an increase in Fmax, which was attributed to conformational rearrangements during co-precipitation that moderately increased the polarity around tryptophan residues. These observations suggested that increasing the proportion of EWP enhanced intermolecular interactions and promoted structural rearrangement in the co-precipitated proteins. This was consistent with previous reports showing that co-precipitation induced stronger protein–protein interactions and fluorescence quenching in SPI-WP systems [1].

### 3.4. Fourier Transform Infrared Spectroscopy (FTIR)

**Figure 4:**
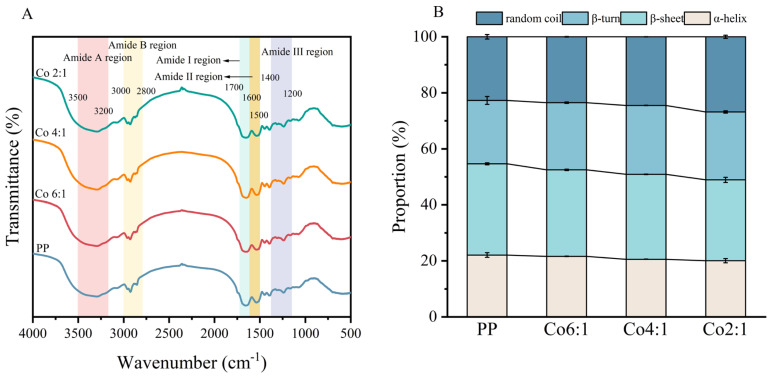
FTIR spectra (**A**) and secondary structure contents (**B**) of PP and PP-EWP co-precipitated proteins at different ratios.

FTIR enables the analysis of protein structural features through the vibrational characteristics of chemical bonds and can be used to monitor changes in secondary structure, where shifts in specific absorption bands can provide evidence for conformational transitions [15]. As displayed in Figure 4A, the amide A band (3200–3500 cm^−1^) was associated with N-H stretching vibrations influenced by hydrogen bonding [7]. Compared with PP, the amide A band of PP-EWP co-precipitated proteins exhibited a slight red shift, suggesting the involvement of stronger hydrogen bonding interactions. Simultaneously, the reduction in absorption intensity could be attributed to partial unfolding of the protein structure and the resulting restriction of N-H group vibrations. The absorption bands observed at approximately 2927 cm^−1^ and 2925 cm^−1^ were associated with C–H_2_ stretching vibrations, whose intensity correlated with the quantity of hydrophobic groups [20]. The decreased absorption intensity in PP-EWP co-precipitated proteins compared with PP suggested reduced exposure of hydrophobic groups on the protein surface.

The amide I band is located within the wavenumber range of 1600–1700 cm^−1^ and is typically used to analyze the secondary structure of proteins. It is primarily divided into the following four structural elements: *β*-sheet (1600–1640 cm^−1^), random coil (1640–1650 cm^−1^), *α*-helix (1650–1660 cm^−1^), and *β*-turn (1660–1700 cm^−1^) [23]. Previous studies have suggested that a decrease in *α*-helix content is associated with protein unfolding, whereas *β*-sheet structures are generally considered to contribute to structural stability [15,31]. As shown in Figure 4B, PP exhibited the highest *α*-helix content (22.09%) and *β*-sheet content (32.57%), indicating a relatively compact native structure. With increasing proportions of EWP, the *α*-helix and *β*-sheet contents of PP-EWP co-precipitated proteins decreased, whereas the contents of random coil and *β*-turn increased. These changes suggested that the protein molecules underwent partial unfolding and structural rearrangement, leading to a relatively more flexible conformation. It has been reported that *α*-helix and *β*-sheet structures contribute to protein structural order and compactness, whereas random coil structures confer conformational flexibility [7]. These results suggested that the incorporation of EWP increased the structural flexibility of the co-precipitated proteins through secondary structure rearrangement.

### 3.5. Microstructural Characteristics of Proteins

**Figure 5:**
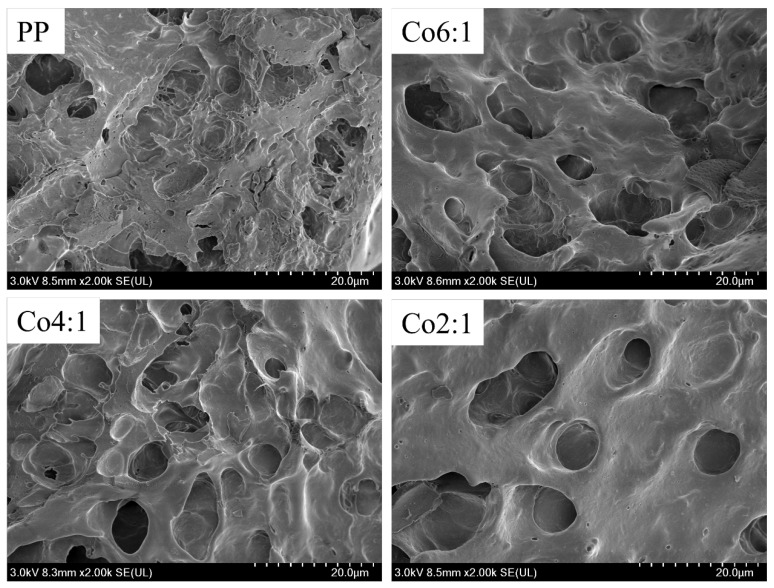
Microstructure of PP and PP-EWP co-precipitated proteins at different ratios, ×2000 magnification.

SEM observations showed that co-precipitation modified the morphology of the proteins (Figure 5). PP exhibited a rough microstructure, whereas the surfaces of the co-precipitated proteins became progressively smoother with increasing EWP proportions, which was related to the hydrophilic nature of EWP molecules [12]. During co-precipitation, the introduction of EWP reduced the surface exposure of hydrophobic regions in PP and promoted intermolecular interactions between the two proteins. Consistent with this, FTIR analysis showed a slight red shift in the amide A band (Figure 4A), suggesting the possible involvement of hydrogen bonding in the co-precipitated system. Particle size analysis (Figure 2A) further showed a gradual decrease in the size of the co-precipitated proteins with increasing EWP proportion. The smaller protein aggregates were more likely to undergo a relatively uniform arrangement during co-precipitation, which contributed to a more uniform and compact microstructure.

### 3.6. SDS-PAGE

**Figure 6:**
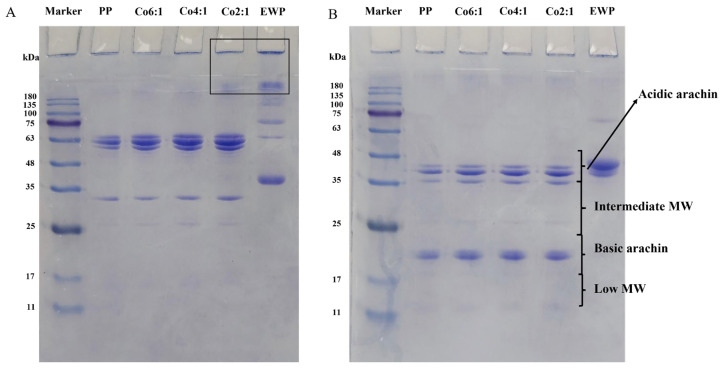
SDS-PAGE patterns of PP, EWP, and PP-EWP co-precipitated proteins at different ratios under non-reducing ((**A**), without *β*-ME) and reducing ((**B**), with *β*-ME) conditions. The black rectangle indicates the high-molecular-weight aggregates observed in Co2:1 and EWP under non-reducing conditions.

As shown in Figure 6B, PP showed four distinct molecular weight regions: the acidic arachin region (MW 38–49.9 kDa), the medium MW region (23–37.9 kDa), the basic arachin region (MW 18–22.9 kDa), and the low MW protein region (14–17.9 kDa) [33]. In the absence of *β*-ME (Figure 6A), Co2:1 and EWP exhibited more intense bands in the high-MW region, suggesting the presence of more high-molecular-weight protein aggregates. In the absence of *β*-ME, the disulfide bonds in protein molecules were not cleaved, whereas the addition of *β*-ME led to the dissociation of subunits stabilized by disulfide bonds from the protein aggregates [34]. In the presence of *β*-ME, the PP-EWP co-precipitated proteins exhibited greater band diffusivity at lower MW, which was consistent with the apparent molecular-weight reduction caused by the reductive cleavage of disulfide bonds. This observation suggested that disulfide bonds were involved in maintaining the aggregated structures of the co-precipitated proteins and may have participated in intermolecular interactions between PP and EWP.

In both the presence and absence of *β*-ME, all co-precipitated samples exhibited stronger protein band intensities than the PP control. This phenomenon was associated with the improved solubility of the proteins after co-precipitation. EWP contains abundant polar groups [12]. Its increased proportion in the co-precipitation system reduced the surface exposure of hydrophobic regions in PP and improved the dispersibility of the co-precipitated proteins, allowing more protein molecules to remain migratable under electrophoretic conditions. Therefore, under the same loading volume, the co-precipitated samples showed darker bands than the PP sample. This result was consistent with the improved solubility and dispersibility of the co-precipitated proteins. In addition, the distinct band patterns of the co-precipitated proteins, compared with those of PP and EWP alone, suggested the occurrence of interactions between the two proteins during co-precipitation.

### 3.7. Molecular Docking Analysis

**Figure 7:**
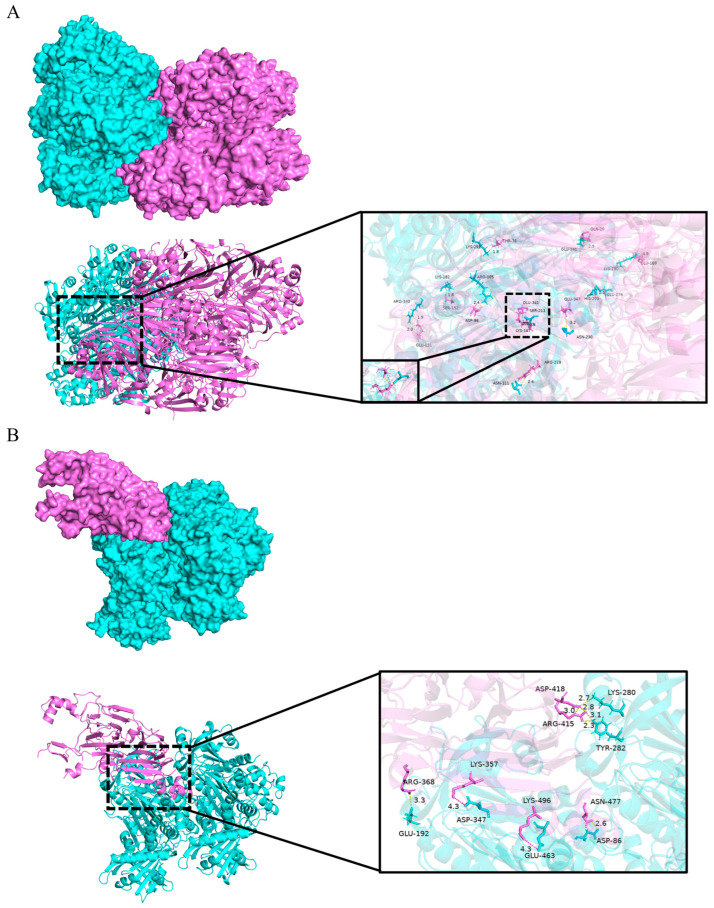
Molecular docking results of ovalbumin with the main regions of conarachin (**A**) and arachin (**B**). Cyan represents ovalbumin, and wine-red represents conarachin in (**A**) and arachin in (**B**). In the enlarged interaction panels, yellow dotted lines indicate hydrogen bonds, and purple dotted lines indicate salt bridges.

PP is primarily composed of arachin and conarachin [35]. The main component of EWP is ovalbumin (approximately 54%) [11]. Therefore, the major regions of arachin and conarachin from PP were selected for molecular docking with ovalbumin. As shown in Figure 7A, conarachin was predicted to form hydrogen bonds with ARG-340, LYS-182, ARG-105, LYS-291, ASN-111, GLU-341, ASN-230, and SER-213 of ovalbumin through its residues GLU-131, SER-152, ASP-96, THR-76, ARG-219, GLN-20, GLU-347, and LYS-187. As illustrated in Figure 7B, the interaction between arachin and ovalbumin was predicted to involve hydrogen bonds and ionic interactions, where ARG-368, ASN-477, ASP-418, and ARG-415 of arachin formed hydrogen bonds with GLU-192, ASP-86, LYS-280, and TYR-282 of ovalbumin, respectively. The residues LYS-357 and LYS-496 of arachin formed salt bridges with ASP-347 and GLU-463 of ovalbumin, respectively. Furthermore, the proximity of these hydrogen-bonded residues enhanced the strength of non-covalent bonds and stabilized the spatial conformation of the co-precipitated proteins [7]. These results were consistent with the FTIR results (Figure 4A) and provided theoretical support for the possible involvement of hydrogen bonding in the interactions between PP and EWP. Notably, conarachin appeared to show a stronger interaction tendency with ovalbumin than arachin in the docking simulation.

### 3.8. Gel Strength and WHC

Gel strength is an important indicator of the integrity and intermolecular cross-linking of the gel network formed by PP-EWP co-precipitated proteins. As shown in Table 1, GCo2:1 exhibited the highest gel strength (911.94 g). The PP gel exhibited relatively low gel strength, which was associated with its low solubility and large particle size, both of which are unfavorable for the formation of a stable gel network. This was consistent with previous observations in other co-precipitated protein gel systems, where weaker and less stable gel networks were also associated with lower solubility and larger particle size [1]. Significant increases in gel strength (*p* < 0.05) were observed at GCo4:1 and GCo2:1 ratios as the proportion of EWP increased. This enhancement was partly attributed to the abundant sulfhydryl groups in EWP, which facilitate disulfide bond formation during gelation [13]. The involvement of disulfide bonds is generally considered beneficial for strengthening gel networks and improving gel texture [36].

WHC is an important physical property of protein gels and is defined as the ability of a gel to retain water within its three-dimensional network under external force [22]. The strength of intermolecular interactions affects the integrity of the three-dimensional network structure, and WHC is closely related to the textural properties of the gel. As shown in Table 1, the incorporation of EWP improved the water retention capacity of the gel network. Previous studies have suggested that, at pH 7.0, EWP undergoes more extensive unfolding before aggregation because of its increased net negative charge, thereby favoring the formation of gels with higher WHC [11]. Meanwhile, the SH_F_ results (Figure 1B) indicated that the incorporation of EWP progressively increased the SH_F_ in PP-EWP co-precipitated proteins, which facilitated the formation of additional disulfide bonds during gelation. This enhanced cross-linking contributed to the formation of a more compact gel network, thereby improving the retention of water within the gel matrix [37].

### 3.9. Contribution of Intermolecular Forces to Gel Formation

Intermolecular forces in protein gels, including ionic bonds, hydrogen bonds, hydrophobic interactions, and disulfide bonds, are considered critical forces for maintaining gel structural stability [21]. The relative contributions of these forces can be estimated by measuring protein solubility in different chemical solvents, which is a commonly used approach to evaluate intermolecular interactions during gel formation [22]. As shown in Table 2, hydrophobic interactions and disulfide bonds were the predominant forces, whereas hydrogen bonds and ionic bonds accounted for relatively smaller proportions. Similarly, a previous study also reported that non-covalent bonds (including hydrophobic interactions) and disulfide bonds served as the main intermolecular forces in co-precipitated proteins [28].

During the heating process of gel formation, hydrophobic groups may become exposed and participate in intermolecular association [38]. PP showed a greater tendency for hydrophobic group exposure, which led to relatively stronger hydrophobic interactions. In contrast, the hydrophobic interactions in co-precipitated proteins decreased due to the masking of the hydrophobic regions of PP by the hydrophilic components of EWP. This aligned with the results for surface hydrophobicity (Figure 3A). With increasing EWP proportions, the disulfide bond content in the gels significantly increased (*p* < 0.05), which was attributed to the conversion of more sulfhydryl groups into disulfide bonds during gelation [38]. Furthermore, EWP contains abundant sulfhydryl groups that are also converted into disulfide bonds during gelation [13]. These findings were consistent with the gel strength results (Table 1). A gradual increase in ionic bonds was observed with progressively higher EWP proportions. This was related to structural rearrangement during co-precipitation and gelation, which increased the accessibility of charged groups and favored electrostatic interactions within the gel network. The formation of heat-induced gels involves the disruption and reformation of hydrogen bonds. Initially, hydrogen bonds stabilize protein–water interactions, whereas during gelation, their partial disruption reduces protein–water binding and promotes stronger protein–protein associations, thereby facilitating intermolecular cross-linking and gel network formation.

### 3.10. Analysis of the Protein Gels Microstructure

**Figure 8:**
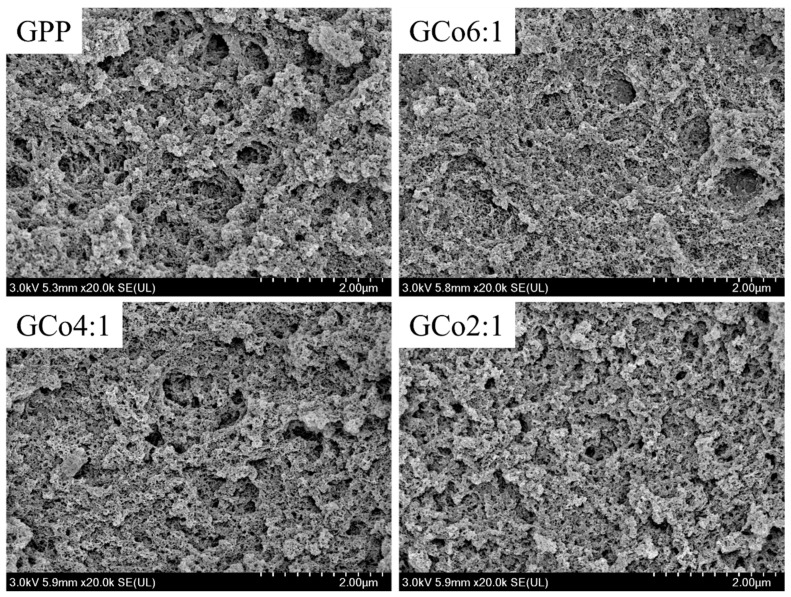
Microstructure of PP and PP-EWP co-precipitated protein gels at different ratios, ×20,000 magnification.

SEM provides information on the internal structure of gels and helps interpret their functional properties [20]. The microstructures of the gels are presented in Figure 8. The PP gel exhibited large aggregates and relatively thick protein strands surrounding the pores. During heat-induced gel formation, the hydrophobic groups within PP may become exposed, promoting excessive aggregation and resulting in a coarse and heterogeneous gel structure. Previous research has demonstrated that excessive protein aggregation can lead to a more fragile gel network [1]. These observations suggested that PP alone tended to form a relatively fragile and unstable three-dimensional network during gelation. In contrast, all PP-EWP co-precipitated proteins formed more compact gel structures. Furthermore, a previous study reported that SPI-WP co-precipitates with dense gel networks exhibited higher WHC [1]. This was consistent with the results of this study. Notably, more prominent aggregates were observed in GCo4:1 and GCo2:1 than in GCo6:1 (Figure 8). This phenomenon was related to the stronger intermolecular cross-linking associated with the increased EWP proportion. Ovalbumin and other components in EWP participate in the formation of a stronger gel network through intermolecular disulfide bond formation during heat-induced gelation [11]. This interpretation was consistent with the increase in disulfide bond content shown in Table 2 as the proportion of EWP increased. In the GCo2:1 system, the higher proportion of EWP provided more opportunities for effective intermolecular cross-linking, thereby promoting more pronounced aggregation and network rearrangement. These structural features contributed to the improved mechanical strength of the three-dimensional network, which was consistent with the gel strength results shown in Table 1.

### 3.11. Discussion

**Figure 9:**
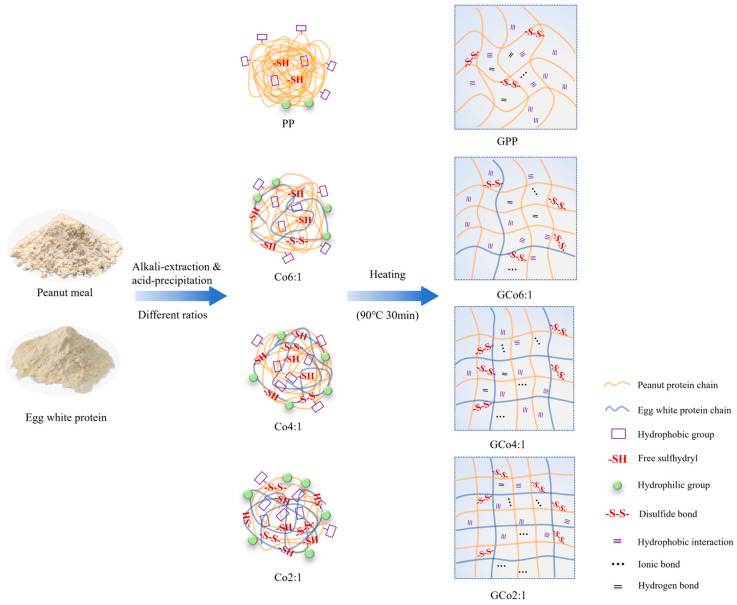
Schematic diagram of the proposed structural rearrangement and heat-induced gelation mechanisms of PP and PP-EWP co-precipitated proteins at different ratios.

A schematic diagram illustrating the mechanism by which co-precipitation improves the gel properties of PP is presented in Figure 9. During co-precipitation, pH adjustment induced partial unfolding of the compact PP structure, resulting in the exposure of previously buried hydrophobic groups. Meanwhile, the abundant hydrophilic groups in EWP partially shielded these exposed hydrophobic regions, thereby reducing surface hydrophobicity (Figure 3A) and suppressing excessive aggregation. As a consequence, the co-precipitated proteins exhibited improved dispersion characteristics, as reflected by smaller particle sizes (Figure 2A), increased absolute zeta potential (Figure 2B), and enhanced solubility (Figure 1A). In addition to these changes, co-precipitation also modified the reactivity of the PP-EWP system. The incorporation of EWP progressively increased the SH_F_ of the co-precipitated proteins (Figure 1B), indicating a greater availability of reactive thiol groups for subsequent intermolecular interactions. FTIR analysis (Figure 4B) revealed a decrease in *α*-helix content and an increase in random coil content, reflecting partial unfolding of the protein structure. These conformational changes likely facilitated molecular rearrangement, which may promote intermolecular association. In agreement with this, differences in protein band patterns under both non-reducing (Figure 6A) and reducing (Figure 6B) conditions in SDS-PAGE supported the involvement of disulfide bonds in the PP-EWP system, while molecular docking analysis (Figure 7) further supported the involvement of hydrogen bonding and ionic interactions in intermolecular association. These results collectively indicate that co-precipitation effectively modified both the structural characteristics and intermolecular interaction patterns of the PP-EWP system.

During heat-induced gelation, these structural changes, together with intermolecular interactions, facilitated the formation of the final gel network. The increased availability of sulfhydryl groups promoted the formation of disulfide bonds upon heating, while non-covalent interactions, including hydrophobic interactions, hydrogen bonds, and ionic interactions, contributed to network stabilization. Compared with PP alone, which tended to undergo excessive and disordered aggregation due to exposed hydrophobic groups, the presence of EWP modulated this process by limiting uncontrolled aggregation and promoting more uniform intermolecular cross-linking, thereby facilitating the formation of a more homogeneous network structure. Consequently, a denser and more continuous three-dimensional gel network was formed. The combined effects of improved dispersion, structural rearrangement, and enhanced intermolecular interactions contributed to the improved gel strength and WHC of PP-EWP co-precipitated protein gels, with the most pronounced improvement observed at the PP:EWP ratio of 2:1 within the tested range (Table 1). From a practical perspective, protein co-precipitates have been regarded as promising food ingredients because of their tunable functional and nutritional properties [8]. Accordingly, in practical applications, the proportion of EWP incorporated into PP-EWP co-precipitated protein systems should be determined according to target product characteristics and formulation feasibility. Consumer-related considerations, such as labeling requirements and acceptance of egg-derived ingredients, should also be taken into account.

## 4. Conclusions

In this study, PP-EWP co-precipitated proteins were prepared to regulate the structure and heat-induced gelation behavior of PP. Co-precipitation with EWP induced secondary structure rearrangement of PP, as reflected by decreased *α*-helix and *β*-sheet contents and increased random coil and *β*-turn contents. The reduced surface hydrophobicity and increased SH_F_ suggested that excessive hydrophobic aggregation was weakened and more reactive groups became available for subsequent gelation. During heat-induced gelation, disulfide bonds and non-covalent interactions contributed to the formation of a denser and more continuous gel network, thereby improving gel strength and WHC. Within the tested range, the improvement in gel performance became more pronounced as the proportion of EWP increased. These findings indicate that co-precipitation with EWP is an effective strategy for modifying PP structure and improving its gelation performance, providing a theoretical basis for the application of PP in protein-rich foods requiring desirable texture and water retention. Considering that co-precipitation altered protein conformation and gel network structure, future studies should further evaluate the digestibility, allergenicity-related properties, and nutritional implications of PP-EWP co-precipitated proteins.

## Figures and Tables

**Table 1:** Gel strength and WHC of PP and PP-EWP co-precipitated protein gels at different ratios.

Samples	Gel Strength (g)	WHC (%)
GPP	429.30 ± 1.71 ^c^	56.78 ± 0.83 ^d^
GCo6:1	417.29 ± 1.54 ^c^	67.93 ± 0.27 ^c^
GCo4:1	580.54 ± 16.80 ^b^	76.61 ± 1.08 ^b^
GCo2:1	911.94 ± 0.58 ^a^	85.53 ± 0.94 ^a^

Results are expressed as mean value ± standard deviation. Mean values followed by different letters are significantly different at *p* < 0.05.

**Table 2:** Intermolecular forces of PP and PP-EWP co-precipitated protein gels at different ratios.

Samples	Hydrophobic Interaction (%)	Disulfide Bond (%)	Hydrogen Bond (%)	Ionic Bond (%)
GPP	54.67 ± 1.12 ^a^	21.40 ± 0.79 ^d^	17.26 ± 0.65 ^a^	6.67 ± 0.24 ^d^
GCo6:1	50.46 ± 0.49 ^b^	26.42 ± 0.73 ^c^	15.59 ± 0.41 ^b^	7.52 ± 0.42 ^c^
GCo4:1	47.24 ± 1.07 ^c^	28.85 ± 0.53 ^b^	14.59 ± 0.40 ^c^	9.32 ± 0.46 ^b^
GCo2:1	41.00 ± 0.98 ^d^	34.13 ± 0.37 ^a^	13.56 ± 0.17 ^d^	11.31 ± 0.30 ^a^

Results are expressed as mean value ± standard deviation. Mean values followed by different letters are significantly different at *p* < 0.05.

## Data Availability

The original contributions presented in this study are included in the article. Further inquiries can be directed to the corresponding author.
